# The efficacy and safety of PD-1/PD-L1 immune checkpoint inhibitors in treating advanced urothelial cancer: a meta-analysis of clinical trials

**DOI:** 10.18632/aging.203429

**Published:** 2021-08-23

**Authors:** Fei Li, Yu Wang, Kunfeng Xie, Yunze Fang, Yuejun Du, Lina Hou, Wanlong Tan

**Affiliations:** 1 Department of Urology, Nanfang Hospital, Southern Medical University, Guangzhou, Guangdong 510515, PR China; 2 Department of Healthy Management, Nanfang Hospital, Southern Medical University, Guangzhou, Guangdong 510515, PR China

**Keywords:** urothelial cancer, immunotherapy, efficacy, safety, meta-analysis

## Abstract

Survival outcomes in advanced urothelial cancer (UC) are dismal. Over the past years, immunotherapy remains an evolving treatment modality for these patients. This meta-analysis was performed to comprehensively evaluate the efficacy and safety of immune checkpoint inhibitors. For this purpose, 18 clinical trials comprising a total of 3,144 patients were identified from the PubMed database up to September 2020. Overall, the objective response rate (ORR) to PD-1/PD-L1 inhibitors was 0.20 [95% confidence intervals (CI) 0.17–0.23]. Furthermore, the pooled 1-year overall survival (OS) and 1-year progression-free survival (PFS) rates were 0.43 (95% CI 0.33–0.53) and 0.19 (95% CI 0.17–0.21), respectively. The summary rates of any-grade and grade ≥3 adverse events (AEs) were 0.66 (95% CI 0.58–0.74) and 0.13 (95% CI 0.09–0.18), respectively. Among the different subgroups, PD-1/PD-L1 inhibitors elicited a promising ORR in patients with lymph node-only metastasis compared to those with visceral metastasis (0.41 VS. 0.17). Additionally, patients with primary tumor in the lower tract had higher ORR compared to those with primary tumor in the upper tract (0.24 VS. 0.15). Briefly speaking, this immunotherapy protocol showed an encouraging efficacy and acceptable safety profile in the treatment of advanced UC. Moreover, our findings provided potential clinical significance for patients with lymph node-only metastasis or primary tumor in the lower tract. However, these exciting findings need further confirmation.

## INTRODUCTION

Bladder cancer is the 10th most prevalent form of cancer worldwide. Advanced urothelial cancer (UC) has a poor prognosis, and the efficacy of therapeutic options currently available for these patients is limited [1, 2]. Nowadays, platinum is still the first-line chemotherapy for advanced UC [3]. Unfortunately, about 30% of patients with advanced UC are considered to be platinum ineligible because of impaired renal function, comorbidities or other reasons. Thus, developing effective treatment strategies remains quite challenging [4]. In addition, the clinical efficacy of these second-line drugs including vinflunine or taxanes and gemcitabine in the treatment of advanced UC is not ideal, and there is still an urgent need for another effective treatment [5].

In recent years, immunotherapy has become an increasingly promising therapeutic method for advanced UC, with immune checkpoint inhibitors being able to halt immune evasion of cancer cells by preventing programmed cell death protein 1 (PD-1) from binding to its ligand [6]. In the past few years, the U.S. Food and Drug Administration (FDA) have approved 6 immune checkpoint inhibitors (Atezolizumab, Pembrolizumab, Durvalumab, Nivolumab, Avelumab and Tislelizumab) for clinical treatment of patients diagnosed with advanced UC or cisplatin-ineligible, who were previously treated with first-line standard chemotherapy [7].

The efficacy and safety profile of PD-1/PD-L1 inhibitors are the major concern related to immunotherapy. Recently, a meta-analysis conducted by Zhang et al., including studies performed before July 2019, reported that the pooled ORR of immune checkpoint inhibitors was 0.20, and the 1-year OS and 1-year PFS rates were 0.50 and 0.17, respectively. The summary frequencies of any-grade and grade ≥3 AEs were 0.65 and 0.11, respectively [8]. However, 6 other studies on the association between immune checkpoint inhibitors and advanced UC were carried out last year. Thus, we systematically collected available published data and performed an updated meta-analysis to investigate the efficacy and safety of PD-1/PD-L1 inhibitors in the treatment of advanced UC patients. The outcomes were then compared across subgroups stratified by different PD-L1 expression levels, studied drugs, and metastasis or primary tumor locations.

## MATERIALS AND METHODS

### Literature search

We conducted a thorough search of the PubMed database to identify the relevant literature until October 2020, using the following research terms: “metastatic bladder cancer” OR “metastatic urothelial carcinoma” OR “bladder cancer” OR “transitional cell carcinoma” AND “PD-L1” OR “PD-1” OR “immunotherapy” OR “immune checkpoint inhibitor” OR “Pembrolizumab” OR “Atezolizumab” OR “Avelumab” OR “Durvalumab” OR “Tislelizumab” OR “Nivolumab” [8]. The search was focused on human studies, without restriction on language. We also checked for relevant articles and their references to search all eligible literature. Two authors (Y.W. and K.F.X.) independently screened the literature for eligibility and any disagreements were resolved by reaching a consensus.

### Inclusion and excluded criteria

Our meta-analysis included studies which met the following criteria: (1) Patients in all studies were exclusively diagnosed with advanced UC. (2) Patients were treated with PD-1/PD-L1 inhibitors including Atezolizumab, Avelumab Durvalumab, Nivolumab, Pembrolizumab, and Tislelizumab. (3) Studies were all clinical trials assessing PD-1/PD-L1 inhibitors. (4) Studies reported the data on efficacy and safety of PD-1/PD-L1 inhibitors, including following indexes: ORR, 1-year PFS rate, 1-year OS rate, rates of any-grade and grade ≥3 AEs.

The exclusion criteria were as follows: (1) Duplicates; (2) Lack of required data (3) case reports, reviews, ecological analyses and off-topic studies, etc. Besides, if multiple studies were conducted from the same or overlapping cohort, only the most informative one was included.

### Data extraction

Two authors (Y.W. and Y.Z.F.) independently extracted data from the selected studies using a standardized data collection form. Any discrepancy was resolved by discussing and reaching a consensus. The extracted information was: the name of first authors, the publication year, phase of research, use of drugs in the trial, median follow-up time, PD-1/PD-L1 inhibitors used as the first line or the second line, the control group of each clinic trail, dosage of drugs, number of recipients, age of participants, ORR, 1-year PFS rate, 1-year OS rate, rates of any-grade and grade ≥3 AEs.

### Outcomes and quality assessment

The outcome measures included the ORR, 1-year OS rate, 1-year PFS rate, rates of any-grade and grade ≥3 AEs. Quality assessment of the studies was conducted independently by two authors (F.L and Y.J.D) based on the Jadad score by RevMan 5.3 [9], and diverging opinions were resolved by discussion.

### Statistical analysis

In this meta-analysis, we presented evaluation indicators with percentages and its 95% confidence intervals (95% CI). Both the fixed- and random-effects methods were used to estimate the overall association. Statistical heterogeneity among the included studies was measured by the Q-statistic (Statistical significance was set at *P* < 0.05) and *I*^2^ statistic [10, 11]. We calculated the pooled ORR, 1-year PFS rate and 1-year OS rate with 95% CI to evaluate the efficacy profile of PD-1/PD-L1 inhibitors [8]. Similarly, we computed the overall rates for any-grade and grade ≥3 AEs to evaluate the safety of immune check point inhibitors.

Subgroup analyses were conducted to measure possible sources of heterogeneity on the basis of different PD-L1 expression levels, PD-L1/PD-1 inhibitors, studied drugs, visceral or lymph node-only metastasis, and primary tumor in the upper or lower tract. Sensitivity analyses were designed to evaluate the robustness of the results. In addition, Egger’s test and Begg’s were utilized to assess for potential bias [12]. All statistical analyses were performed using RevMan 5.3 (Cochrane Collaboration, Oxford, UK) and the “meta” package in the R software 3.6.0 (R Foundation for Statistical Computing, Vienna, Austria). A two-tailed *P value* <0.05 was considered statistically significant.

## RESULTS

### Literature search results

**Figure 1:**
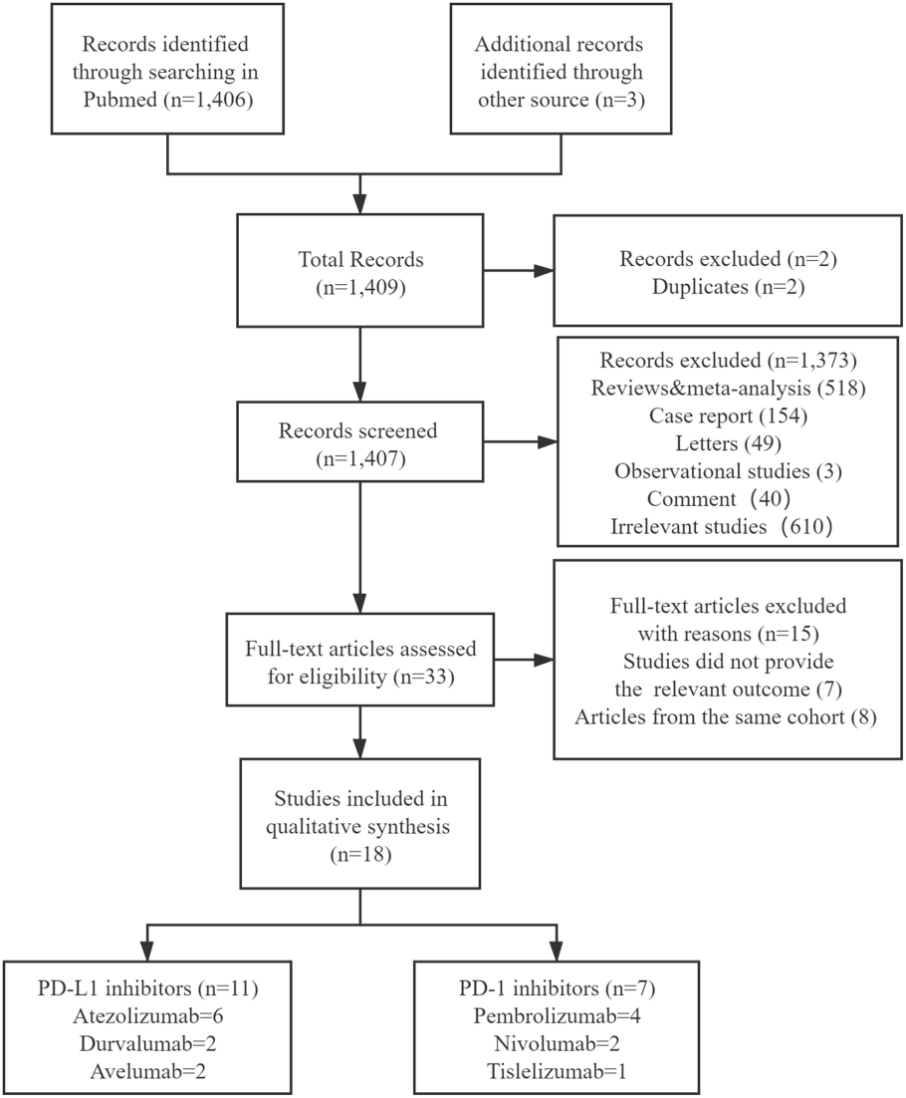
Flow chart of the study selection procedure.

A flow chart of our selection process was illustrated in Figure 1. A total of 1,409 articles were identified after our search. Of those, 33 were considered to be preliminary selected articles for further review after excluding duplicate articles and screening the titles and abstracts to determine their relevance. After a full-text review of the remaining 33 articles, 7 articles were excluded due to the fact that they did not report relevant outcomes. Meanwhile the remaining 8 articles were excluded since their participant cohorts overlapped with other studies. Finally, we included a total of 18 articles in our meta-analysis [12–29] (Figure 1).

### Characteristics of included studies

The characteristics of the included studies were shown in Table 1. The enrolled studies were published between 2014 and 2020. The Jadad score of each included study ranged from 3 to 5, no study received a low-quality Jadad score, validating our selection criteria. Moreover, 11 studies assessed PD-L1 inhibitors (Atezolizumab = 7 [13–19], Durvalumab = 2 [20, 21], Avelumab = 2 [22, 23], whereas 7 articles studied PD-1 inhibitors (Pembrolizumab = 4 [24–27], Nivolumab = 2 [28, 29], Tislelizumab = 1 [30]). Our meta-analysis involved 3,144 patients diagnosed with advanced UC and the medium follow-up time of included studies ranged from 2.3 to 37.8 months (Table 1).

**Table 1:** Baseline characteristics and data of the included studies using PD-1/PD-L1 inhibitors.

**Study**	**Year**	**Journal**	**Phase**	**Intervention**	**Follow-up period (months) (median)**	**Line**	**Dose**
Galsky et al.	2020	The Lancet	Phase III	Atezolizumab	11.8	First	1,200 mg IV q3 weeks
Vuky et al.	2020	Journal of Clinical Oncology	Phase II	Pembrolizumab	11.4	First	200 mg IV q3 weeks
Shen et al.	2020	Journal for ImmunoTherapy of Cancer	Phase I/II	Tislelizumab	8.1	Second	2mg/Kg IV q3 weeks
Nishiyama et al.	2019	Journal of Clinical Oncology	Phase III	Pembrolizumab	14.1	Second	200 mg IV q3 weeks
Petrylak et al.	2018	JAMA Oncology	Phase I	Atezolizumab	37.8	Second	1,200 mg IV q3 weeks
Pal et al.	2018	European Association of Urology	NR	Atezolizumab	17.3	Second	1,200 mg IV q3 weeks
Velde et al.	2018	European Association of Urology	Phase II	Atezolizumab	2.3	Second	1,200 mg IV q3 weeks
Powles et al.	2018	The Lancet	Phase III	Atezolizumab	7.0	First	1,200 mg IV q3 weeks
Patel et al.	2017	The Lancet	Phase I	Avelumab	7.0	Second	10mg/Kg IV q2 weeks
Apolo et al.	2017	Journal of Clinical Oncology	Phase IB	Avelumab	9.9	Second	10mg/Kg IV q2 weeks
Powles et al.	2017	JAMA Oncology	Phase I/II	Durvalumab	16.5	Second	10 mg/kg IV q2 weeks
Sharma et al.	2017	The Lancet Oncology	Phase II	Nivolumab	5.8	Second	3mg/Kg IV q2 weeks
Bellmunt et al.	2017	The New England Journal of Medicine	Phase III	Pembrolizumab	14.1	Second	200 mg IV q3 weeks
Plimack et al.	2017	The Lancet Oncology	Phase IB	Pembrolizumab	13.0	Second	200 mg IV q3 weeks
Rosenberg et al.	2016	The Lancet	Phase II	Atezolizumab	11.7	Second	1,200 mg IV q3 weeks
Massard et al.	2016	Journal of Clinical Oncology	Phase III	Durvalumab	4.3	First/Second	10 mg/kg IV q2 weeks
Sharma et al.	2016	The Lancet Oncology	Phase I/II	Nivolumab	15.2	Second	3mg/Kg IV q2 weeks
Chen et al.	2014	Nature	Phase I	Atezolizumab	4.2	Second	1,200 mg IV q3 weeks

**Study**	**Studied Group No.**	**Control group**	**Control group No.**	**Mean age**	**ORR (%)**	**1-year OS rate (%)**	**1-year PFS rate (%)**	**Any-grade AEs rate (%)**	**Grade ≥3 AEs rate (%)**
Galsky et al.	362	Group A and C^a^	451/400	67 (62–74)	23	NR	NR	93	42
Vuky et al.	370	None	/	74 (34–94)	28.6	46.9	22	67.3	20.8
Shen et al.	22	None	/	63 (55–67)	14	0.3	0.2	NR	NR
Nishiyama et al.	30	chemotherapy	22	72 (51–83)	20	40	13.3	56.7	16.7
Petrylak et al.	95	None	/	66 (36–89)	26	45	NR	67	9
Pal et al.	214	None	/	69 (62–76)	15	NR	NR	45	7
Velde et al.	110	None	/	72 (66–79)	NR	55.5	NR	NR	NR
Powles et al.	467	chemotherapy	182	67 (33–88)	13.4	46.4	NR	69	6
Patel et al.	161	None	/	68 (36–76)	17	NR	NR	67	8
Apolo et al.	44	None	/	68 (63–73)	18.2	54.3	19.1	65.9	6.8
Powles et al.	191	None	/	57 (26–82)	17.8	55	16	60.7	6.8
Sharma et al.	265	None	/	66 (38–90)	19.6	NR	NR	64	18
Bellmunt et al.	270	chemotherapy	272	67 (NR)	21.1	43.9	17	60.9	15
Plimack et al.	27	None	/	70 (44–85)	26	50	16	60	15
Rosenberg et al.	310	None	/	65 (36–86)	15	37	NR	69	16
Massard et al.	61	None	/	66 (34–81)	31	NR	NR	63.9	4.9
Sharma et al.	78	None	/	65.5(31–85)	24.4	46	20.8	81	22
Chen et al.	67	None	/	63 (36–86)	NR	NR	NR	57.4	4.4

^a^Group A (Atezolizumab plus chemotherapy) and group C (placebo plus chemotherapy).

Abbreviations: AE: adverse event; IV: intravenous; NR: not reported; ORR: objective response rate; OS: overall survival; PFS: progression-free survival.

### Efficacy assessment

**Figure 2:**
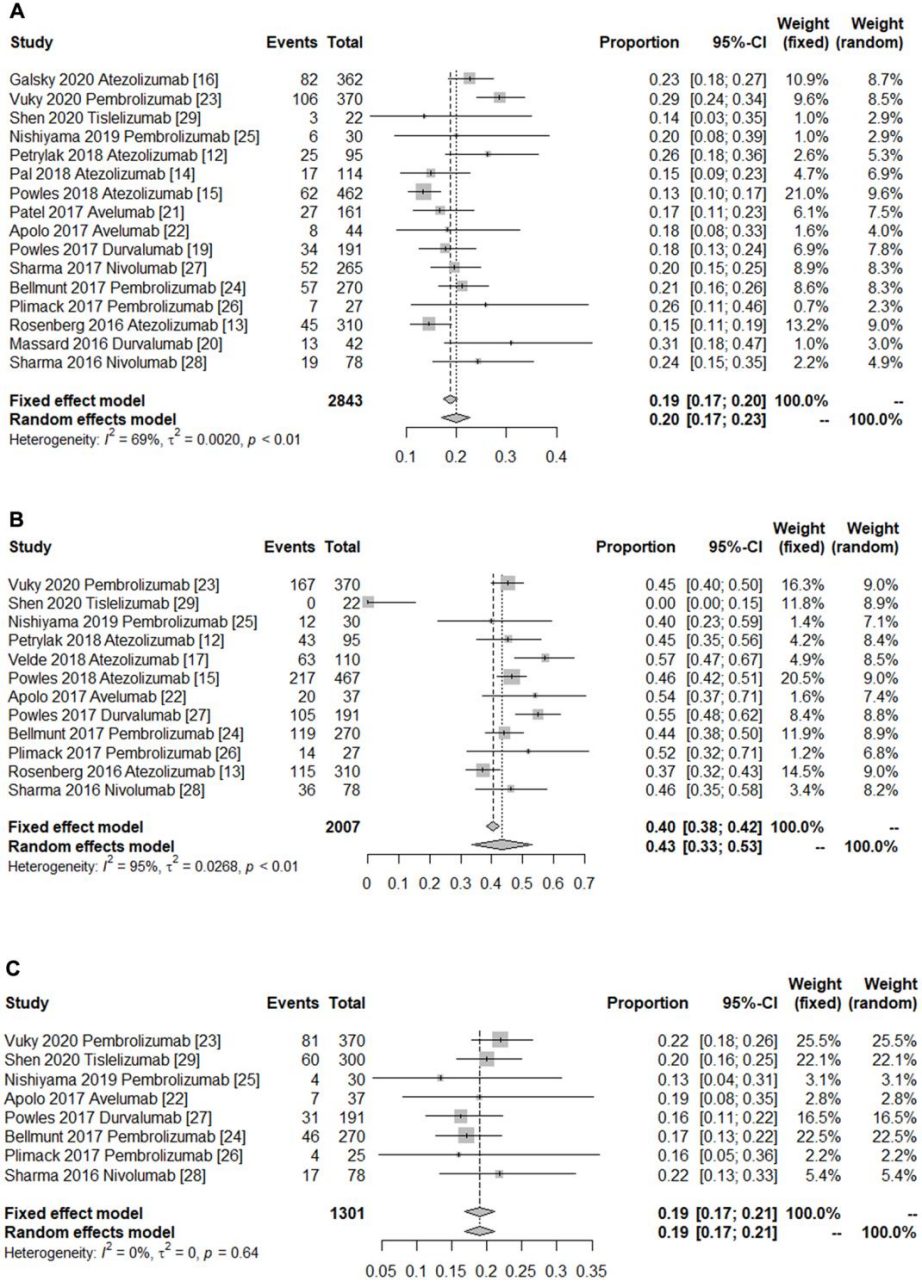
**Forest plot of the efficacy of immune checkpoint inhibitors in treating patients with advanced urothelial cancer.** (**A**) Pooled objective response rate. (**B**) Pooled 1-year overall survival rate. (**C**) Pooled 1-year progress free survival rate. The diamonds represent the pooled indexes. The line crossing the square represents the 95% CI. *I*^2^ indicates the heterogeneity in each subgroup meta-analysis. *P* demonstrates the significance of differences between the subgroups.

We used the pooled ORR, 1-year PFS rate and 1-year OS rate to evaluate the efficacy of PD-1/PD-L1 immune checkpoint inhibitors in treatment of advanced UC. Figure 2 delineates the ORR for advanced UC using the random-effects model based on 16 studies [12–16, 19–29] with a sample size of 2,843 individuals. The pooled ORR was 0.20 (95% CI 0.17–0.23, Figure 2A). The pooled complete response (CR) and partial response (PR) were 0.05 and 0.14, respectively. Furthermore, 12 studies [12, 13, 15, 17, 22–29] were assessed for the 1-year OS rate, with the pooled 1-year OS rate being 0.43 (95% CI 0.33-0.53, Figure 2B). Additionally, 8 studies [22–29] were assessed for the 1-year PFS rate, and the pooled outcome was 0.19 (95% CI 0.17–0.21, Figure 2C). Substantial heterogeneity was observed across studies in terms of ORR (*I*^2^ = 68.7%, *p* < 0.0001, Figure 2A) and 1-year OS rate (*I*^2^ = 95.1%, *p* < 0.0001, Figure 2B), but no indication of heterogeneity was shown in terms of the 1-year PFS rate (*I*^2^ = 0%, *p* = 0.639, Figure 2C) (Table 2).

**Table 2:** The outcomes of the efficacy of PD-1/PD-L1 inhibitors.

**Analysis Specifications**	**Studies**	**Total event**	**Total population**	**Proportion (95% CI)**	***P* Value Heterogeneity**	***P* Egger’s**	***P* Begg’s**
1-year OS rate	12	911	2007	0.43 (0.33–0.53)	<0.0001	0.493	0.593
1-year PFS rate	8	250	1301	0.19 (0.17–0.21)	0.639	0.266	0.298

ORR	16	563	2843	0.20 (0.17–0.23)	<0.0001	0.653	0.195
CR	14	136	2531	0.05 (0.04–0.06)	0.005	0.020	0.502
PR	14	357	2531	0.14 (0.11–0.16)	0.003	0.556	1.000
SD	14	548	2531	0.21 (0.19–0.24)	0.002	0.825	0.584
PD	14	1078	2531	0.42 (0.35–0.48)	<0.0001	0.829	0.661

Drug of study							
PD-L1inhibitor	9	313	1781	0.18 (0.15–0.21)	0.009	0.086	0.251
Atezolizumab	5	231	1343	0.18 (0.14–0.22)	0.004	0.289	0.221
Avelumab	2	35	205	0.17 (0.12–0.22)	0.830	–	–
Durvalumab	2	47	233	0.20 (0.14–0.25)	0.060	–	–
PD-1 inhibitor	7	250	1062	0.23 (0.21–0.26)	0.114	0.560	1.000
Nivolumab	2	71	343	0.21 (0.16–0.25)	0.384	–	–
Pembrolizumab	4	176	697	0.25 (0.22–0.28)	0.149	0.806	1.000

Expression of PD-L1							
PD-L1 (+)	7	134	503	0.26 (0.22–0.29)	0.026	0.093	0.133
PD-L1 (−)	7	63	468	0.12 (0.06–0.17)	0.001	0.162	1.000

Location of metastasis							
Visceral	7	178	1013	0.17 (0.12–0.23)	<0.0001	0.337	0.548
Lymph node-only	7	70	169	0.41 (0.32–0.50)	0.265	0.723	0.649

Location of primary tumor							
Upper-tract	2	16	105	0.15 (0.08–0.21)	0.366	–	–
Lower-tract	2	111	425	0.24 (0.13–0.35)	0.012	–	–

Abbreviations: ORR: objective response rate; OS: overall survival; PFS: progression-free survival; CR: complete response; PR: partial response; SD: stable disease; PD: progressive disease.

We used Begg’s and Egger’s tests to conduct asymmetry tests and measure the publication bias. The Begg’s test did not establish evidence of publication bias after analysis of the ORR (*P* = 0.195), 1-year OS rate (*P* = 0.593) and 1-year PFS rate (*P* = 0.298). Likewise, the Egger’s test did not point out evidence of publication bias with respect to the ORR (*P* = 0.653), 1-year OS rate (*P* = 0.493), and 1-year PFS (*P* = 0.266).

When studies were stratified based on different PD-L1 expression levels, PD-L1 or PD-1 inhibitors, studied drugs, visceral or lymph node-only metastasis and in the upper or lower tract 9 [12–16, 19–22] studies reported the efficacy of PD-L1 inhibitors, with the pooled ORR being 0.18 (95% CI 0.15–0.21, *I*^2^ = 63.3%, *P* = 0.005). Whilst 7 studies [23–29] reported the efficacy of PD-1 inhibitors, and the pooled ORR was 0.23 (95% CI 0.21–0.26, *I*^2^ = 42%, *P* = 0.114). PD-1 inhibitors had a better efficacy profile compared with PD-L1 inhibitors. In fact, Pembrolizumab had a higher ORR (0.25, 95% CI 0.22–0.28, *I*^2^ = 44%, *P* = 0.149) than all the reported immune checkpoint inhibitors. The pooled ORRs of drug subgroups for Atezolizumab, Durvalumab, Nivolumab and Avelumab were 0.18 (95% CI 0.14–0.22, *I*^2^ = 77.2%, *P* = 0.002), 0.20 (95% CI 0.14–0.25, *I*^2^ = 33%, *P* = 0.004), 0.21 (95% CI 0.16–0.25, *I*^2^ = 0%, *P* = 0.384) and 0.17(95% CI 0.12–0.22, *I* = 0%, *P* = 0.830), respectively (Table 2).

**Figure 3:**
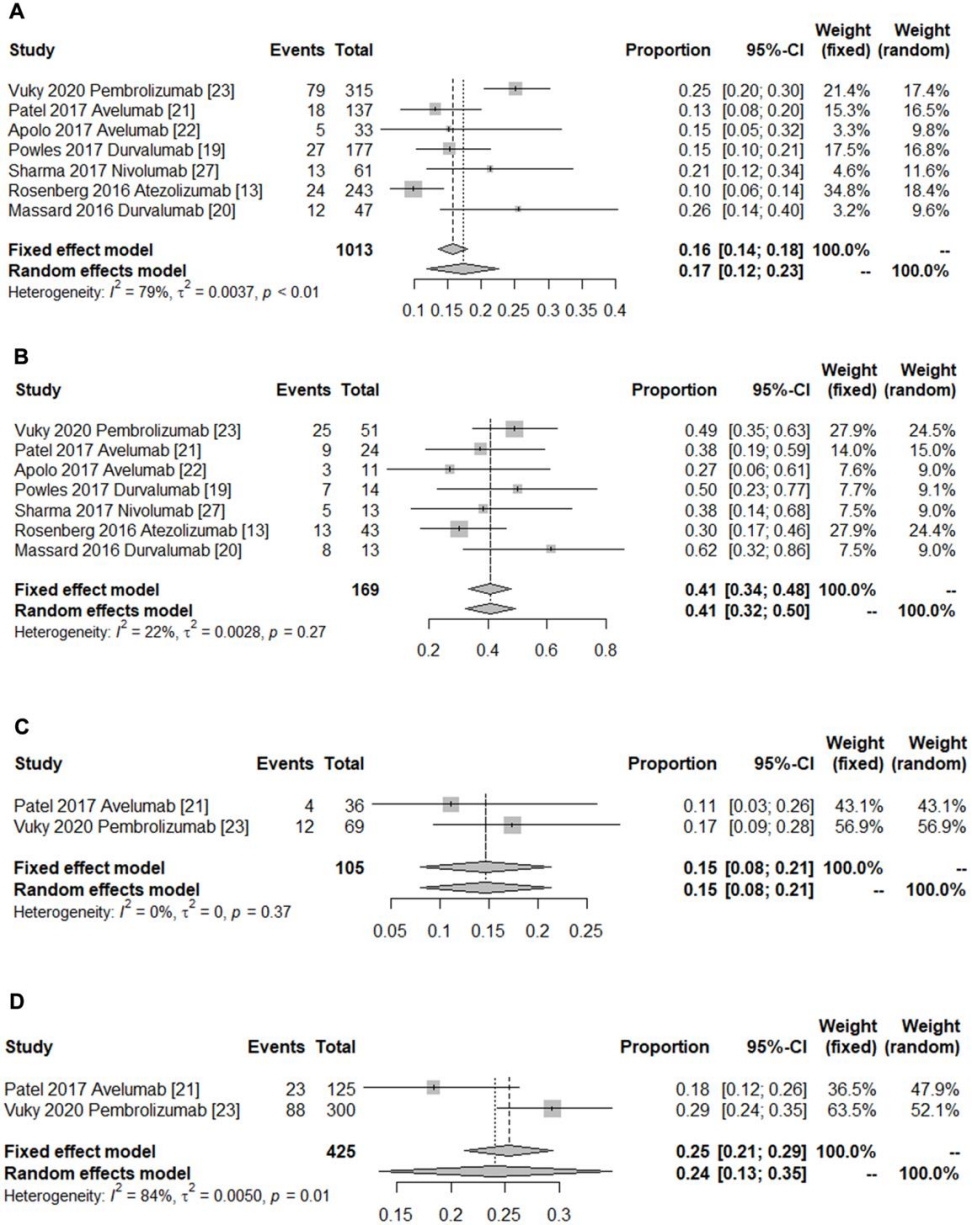
**Forest plot of the subgroup outcomes.** (**A**) Pooled ORR of patients with visceral metastasis. (**B**) Pooled ORR of patients with lymph node only metastasis. (**C**) Pooled ORR of patients with the primary tumor in the upper tract. (**D**) Pooled ORR of patients with the primary tumor in the lower tract. The diamonds represent the pooled indexes. The line crossing the square represents the 95% CI. *I*^2^ indicates the heterogeneity in each subgroup meta-analysis. *P* demonstrates the significance of differences between the subgroups.

The PD-L1 expression levels of included patient tumor samples were evaluated by immunohistochemistry. Subsequently, the group with PD-L1 expression ≥ 1% was denoted as the positive group, and the group with PD-L1 expression < 1% as the negative group. The pooled ORR of the PD-L1-positive group (ORR = 0.26, 95% CI 0.22–0.29, *I*^2^ = 58%, *P* = 0.026) indicated a better efficacy than the PD-L1-negative group (ORR = 0.12, 95% CI 0.06–0.17, *I*^2^ = 74%, *P* = 0.001) (Table 2). Furthermore, patients with lymph node-only metastasis (ORR = 0.41, 95% CI 0.34–0.48, *I*^2^ = 22%, *P* = 0.265) experienced a better efficacy results than those with visceral metastasis (ORR = 0.17, 95% CI 0.12–0.23, *I*^2^ = 79%, *P* < 0.0001) (Figure 3A and 3B). Moreover, the overall ORR for studies with the primary tumor located in the lower tract was 0.24 (95% CI 0.12–0.23), with variability detected (*p*-value for heterogeneity = 0.012, *I*^2^ = 84%). There was less evidence of heterogeneity in studies with the primary tumor located in the upper tract (ORR = 0.15, 95% CI 0.08–0.21, *p-*value for heterogeneity = 0.366, *I*^2^ = 0%) (Figure 3C and 3D).

### Safety assessment

**Figure 4:**
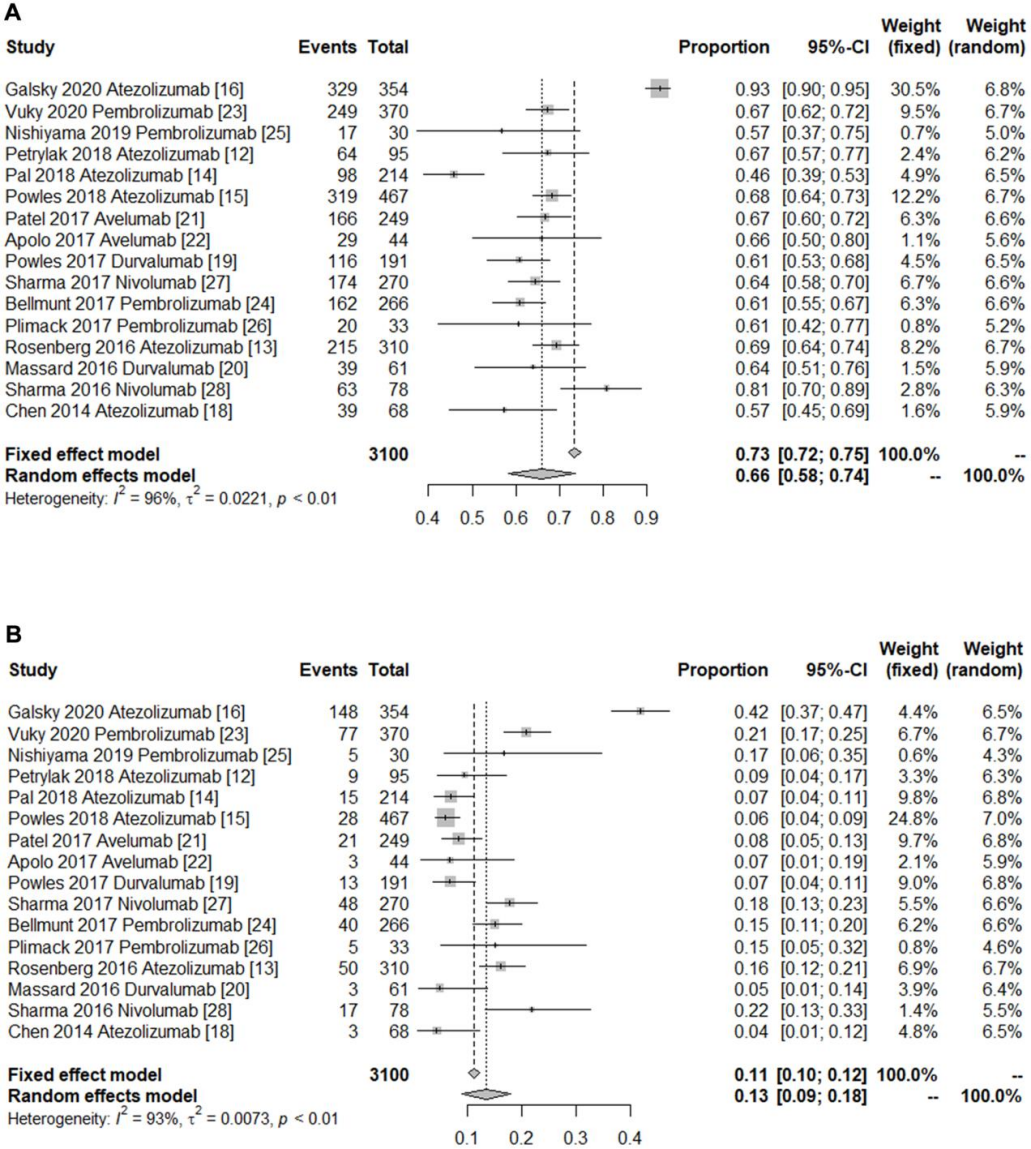
**Forest plot of the safety of immune checkpoint inhibitors in treating patients with advanced urothelial cancer.** (**A**) Pooled any-grade adverse events rate. (**B**) Pooled grade ≥3 adverse events rate. The diamonds represent the pooled indexes. The line crossing the square represents the 95% CI. *I*^2^ indicates the heterogeneity in each subgroup meta-analysis. *P* demonstrates the significance of differences between the subgroups.

The rates of any-grade and grade ≥3 AEs were used to gauge the safety of PD-1/PD-L1 inhibitors in the treatment of metastatic UC. The pooled rates of any-grade and grade ≥3 AEs rates are presented in Figure 4. The summary outcomes for any-grade and grade ≥3 AEs were 0.66 (95% CI 0.58–0.74) and 0.13 (95% CI 0.09–0.18), respectively. Obvious heterogeneity was found in the pooled estimation of the rate of any-grade AEs (*I*^2^ = 95.7%, *P <* 0.0001) and grade ≥3 AEs (*I*^2^ = 93.5%, *P* < 0.0001). Thence, subgroup analysis based on PD-L1/PD-1 inhibitors was performed to explore the sources of heterogeneity. The significant evidence of publication bias was not indicated by the Egger’s and Begg’s tests.

Overall, the pooled rate of any-grade AEs in the PD-1 group (0.66, 95% CI 0.60–0.72 *I*^2^ = 69%, *P* = 0.007) was similar to that in the PD-L1 group (0.66, 95% CI 0.55–0.77, *I*^2^ = 97%, *P* < 0.0001). Interestingly, the summary rate of grade ≥3 AEs in the PD-1 group (0.18, 95% CI, 0.16–0.20, *I*^2^ = 0%, *P* = 0.478) was significantly higher than the PD-L1 group’s (0.11, 95% CI 0.05–0.17 *I*^2^ = 95.1%, *P* < 0.0001) (Table 3). Thus, PD-1/PD-L1 immune checkpoint inhibitors have an acceptable safety outcome.

**Table 3:** The outcomes of the any-grade and ≥3 grade AEs rates of PD-1/PD-L1 inhibitors.

**Analysis Specifications**	**Studies**	**Total event**	**Total population**	**Proportion (95% CI)**	***P* Value Heterogeneity**	***P* Egger’s**	***P* Begg’s**
Any-grade AEs	16	2099	3100	0.66 (0.58–0.74)	<0.0001	0.013	0.964
PD-L1 inhibitor	10	1414	2053	0.66 (0.55–0.77)	<0.0001	0.060	0.474
PD-1 inhibitor	6	685	1047	0.66 (0.60–0.72)	0.007	0.910	0.452

Grade ≥3 AEs	16	485	3100	0.13 (0.09–0.18)	<0.0001	0.129	0.300
PD-L1 inhibitor	10	293	2053	0.11 (0.05–0.17)	<0.0001	0.379	0.474
PD-1 inhibitor	6	192	1047	0.18 (0.16–0.20)	0.478	0.950	0.707

Abbreviation: AE: adverse event.

## DISCUSSION

Advanced UC patients have a poor prognosis. Currently, platinum-based drugs are the therapeutic mainstay for these patients and there has been a lack of effective second-line drugs [31]. Patients with advanced UC still have a lack of effective treatment regimens to slow the disease’s progression long enough for the development of immunotherapy strategy [32, 33]. In recent years, PD-1/PD-L1 immune checkpoint inhibitors remains an evolving treatment modality for advanced UC [34]. To date, FDA has approved 6 immune checkpoint inhibitors for the treatment of advanced UC patients who were previously treated with standard chemotherapy and for those ineligible to the standard chemotherapy.

To make a further analysis of the safety and efficacy of PD-1/PD-L1 inhibitors in treating advanced UC, we performed an up-to-date meta-analysis. In this updated meta-analysis, 18 studies comprising a total of 3, 144 patients diagnosed with advanced UC were included to explore the efficacy and safety of PD-1/PD-L1 inhibitors in the treatment of these patients. Overall, the average ORR for PD-1/PD-L1 inhibitors was 0.20 (95% CI 0.17–0.23, Figure 2A). Furthermore, the pooled 1-year OS and 1-year PFS rates were 0.43 and 0.19, respectively. Thus, PD-1/PD-L1 immune checkpoint inhibitors elicited promising efficacy (Table 2). The underlying mechanism of action of PD-1/PD-L1 immune checkpoint inhibitors in the treatment of advanced UC could be the fact that PD-1/PD-L1 antibodies prevents the immune escape of tumor cells by blocking the binding of PD-1 on T cells to its ligand on tumor cells. The rates of any-grade and grade ≥3 AEs were used to evaluate the drugs’ safety profiles. The overall rate of any-grade AEs did not demonstrate a statistically significant difference in the PD-1 group compared with PD-L1 group. Noticeably, the pooled rates of grade ≥3 AEs in the PD-1 and PD-L1 groups were 0.18 and 0.11, respectively. However, the related mechanism for this finding is unclear.

Substantial heterogeneity was detected in our meta-analysis due to different PD-L1 expression levels, PD-L1/PD-1 inhibitors, studied drugs, visceral or lymph node-only metastasis, and either in the upper or lower tract. We conducted subgroup analyses to investigate the sources of the observed heterogeneity across studies. A large part of the detected heterogeneity may be explained by stratified analysis, which is based on differences in interventions across various studies, locations of metastases and primary tumors. Notwithstanding, we have confirmed an absence of significant publication bias in this meta-analysis either with the Begg’s tests for each study. In addition, our sensitivity analyses revealed similar and robust results.

The research on PD-L1/PD-1 inhibitors has been receiving an increasing amount of attention over the recent years. In 2019, Zhang et al. published a meta-analysis consisting of clinical trials published until July 2019 [8]. The overall ORR was 0.20. However, 6 additional studies [17–19, 24, 26, 30] on this topic have been published between 2019 and 2020. Therefore, an updated meta-analysis was performed to ascertain the efficacy and safety of immune checkpoint inhibitors. Overall, the pooled ORR was 0.20, and the efficacy and safety profiles were similar to those reported in the meta-analysis performed by Zhang et al. Nonetheless, among the subgroup analyses stratified by the location of metastasis or primary tumor, PD-1/PD-L1 inhibitors produced encouraging ORR in advanced UC patients with lymph node-only metastasis compared to those with visceral metastasis (0.41 VS. 0.17). Furthermore, patients with primary tumors situated in the lower tract had higher ORR compared to those with primary tumors in the upper tract (0.24 VS. 0.15). The findings observed from those two subgroups could have clinical guiding significance for the treatment of advanced UC by PD-1/PD-L1 inhibitors.

This study contains several important strengths that have been briefly mentioned below. This is an updated systematic epidemiologic assessment of the safety and efficacy of PD-1/PD-L1 inhibitors in treating advanced UC patients. Our summary analysis of 18 studies involving 3, 144 patients with advanced UC provides a more stable association and reliable estimation. Furthermore, the findings observed in subgroup analyses grouped by location of metastasis or primary tumor have a promising benefit for the clinical management of those patients.

Withal, there are several potential limitations in our current study that need to be taken into account when interpreting the results. First and foremost are the limitations inherent to the majority of included studies, which were prone to have potential performance bias because most of them were different phase of clinical trials, and a larger number of RCTs on PD-1/PD-L1 inhibitors in treating advanced UC patients have not been conducted. Secondly, substantial heterogeneity was observed in this present analysis; although numerous subgroup analyses were conducted, the possible sources of heterogeneity were not identified. Thirdly, the included studies had various classifications of PD-L1 expression levels by different staining cut-off values, which might have an impact on the patient populations and mislead the true summary estimation. In summary, this updated meta-analysis not only confirmed the efficacy and safety of PD-1/PD-L1 inhibitors in treating advanced UC patients but also provided potential clinical significance for patients with lymph node-only metastases or primary tumors located in the lower tract. Nevertheless, further investigation mainly via RCTs is needed to confirm these findings.

### Availability of data and materials

All data generated or analyzed during this study are included in the published articles.
